# The Effects of Different Classes of Antihypertensive Drugs on Patients with COVID-19 and Hypertension: A Mini-Review

**DOI:** 10.1155/2022/5937802

**Published:** 2022-01-21

**Authors:** Farnoosh Nozari, Nasrin Hamidizadeh

**Affiliations:** Molecular Dermatology Research Center, Shiraz University of Medical Sciences, Shiraz, Iran

## Abstract

Hypertension is a major risk factor for cardiovascular disease. Previous studies showed that patients with hypertension are at an increased risk of developing severe COVID-19 infection. Therefore, proper blood pressure control in hypertensive patients with COVID-19 is of great importance. In this review, we discussed the effects of different classes of antihypertensive drugs on patients with hypertension and COVID-19.

## 1. Background

Hypertension is a chronic and age-related condition which leads to cardiovascular and renal complications [1]. An increase in the proportion of adults with hypertension was observed from 26.4% in 2000 to 31.1% in 2010 [2].

Effective control of blood pressure reduces the risk of stroke, heart attack, and heart failure [3]. Antihypertensive drugs comprise different classes of chemical compounds used to prevent and treat high blood pressure [4]. The most commonly prescribed antihypertensive drug classes include angiotensin-converting enzyme (ACE) inhibitors, angiotensin II receptor blockers (ARBs), diuretics, calcium channel blockers (CCBs), and beta-blockers [5].

Novel coronavirus disease 2019 (COVID-19), caused by severe acute respiratory syndrome (SARS-CoV-2), is an emerging respiratory infectious disease, which first occurred in Wuhan, the capital of Chinese province Hubei, in December 2019 [6]. COVID‐19 has a wide spectrum of clinical manifestations, ranging from asymptomatic illness to severe pulmonary infections [7].

Patients with hypertension are at increased risk of developing severe COVID-19 infection [8]. Previous studies reported that up to 30% of people with COVID-19 had chronic arterial hypertension, suggesting that hypertension could play an important role in the aggravation of COVID-19 symptoms [9]. Furthermore, hypertensive patients affected by COVID-19 had a higher mortality risk than patients without hypertension [10].

Since hypertension is one of the main risk factors for developing COVID-19, choosing the proper agent for effective control of blood pressure is crucial.

This review focuses on the effects of various available antihypertensive drugs on managing COVID-19 patients with hypertension.

### 1.1. Alpha-Blockers

Alpha-blockers are a class of drugs that reduce vascular smooth muscle contractions and cause vasodilation [5]. However, they are not used as first-line agents in treating high blood pressure [11]. Several side effects, such as orthostatic hypotension, tachycardia, increased risk of syncope, falls, and fracture, are associated with using alpha-blockers [12].

In acute respiratory distress syndrome (ARDS), various stages including hyaline membrane formation in the alveoli, interstitial widening, edema, and fibroblast proliferation were observed [13]. Analyses in the patients with ARDS and pneumonia showed that using alpha-1 adrenergic receptors was associated with a relative risk reduction of 34% for mechanical ventilation and death [14]. Since ARDS caused by COVID-19 results in the typical ARDS pathological changes in the lungs, using alpha-blockers might be beneficial in this setting [13].

### 1.2. Beta-Blockers

Beta-blockers reduce blood pressure by inhibiting catecholamines from binding to beta-adrenergic receptors, causing vasodilation of coronary and peripheral arteries [15]. The most common side effects of beta-blockers are bradycardia, constipation, sexual dysfunction, fatigue, and bronchospasm [15]. Besides, they have shortening effects on QT interval [16].

A study conducted by Vasanthakumar suggested that using beta-blockers could offer several benefits to treat COVID-19, such as the reduction of the SARS-CoV-2 cell entry via downregulation of the angiotensin-converting enzyme 2 (ACE-2), reducing the expression of proinflammatory cytokines, and the reduction of complications, such as pulmonary embolism, ARDS, and septic shock [17].

Bauer et al. showed that beta-blockers were not associated with the severity of COVID-19 [18]. Another study suggested that treatment with beta-blockers significantly reduced the risk of serious outcomes [19]. Reynolds et al. also found a lower chance of testing positive for COVID-19 in the patients taking beta-blockers [20].

### 1.3. Calcium Channel Blockers (CCBs)

CCBs are used as a first-line treatment option alone or in combination with other antihypertensive drugs in the patients with hypertension [21]. CCBs block the movement of calcium into the cells by binding to L-type voltage-gated calcium channels in various organs such as the heart and vascular smooth muscle [22]. A decrease in the intracellular concentration of calcium induces smooth muscle cell relaxation and subsequent blood pressure reduction [23]. Major adverse reactions caused using this group are headaches, flushing, palpitations, peripheral edema, hypotension, atrioventricular block, constipation, and nausea [24].

Zhang et al. reported that CCBs could inhibit SARS-CoV-2 replication after entry in vitro. Furthermore, amlodipine besylate reduced the mortality rate in hypertensive patients [25]. Another study suggested that nifedipine and amlodipine significantly reduced the death rate and the risk of intubation and mechanical ventilation in elderly patients with COVID-19 [26]. Regarding the mortality rate and the length of hospital and intensive care unit (ICU) stay, Nouri-Vaskeh et al. found no significant differences in the administration of losartan or amlodipine in COVID-19 patients with primary hypertension [27].

### 1.4. ACE Inhibitors and ARBs

 Renin-angiotensin-aldosterone system (RAAS) inhibitors, including ACE inhibitors and ARBs, are among the most commonly prescribed drugs to treat high blood pressure [28]. ACE inhibitors play a role to lower blood pressure by blocking the angiotensin-converting enzyme, which leads to a decrease in angiotensin II production and vasodilation. The action mechanism of ARBs is blocking the binding of angiotensin II to the angiotensin-1 (AT1) receptors [5]. The most important adverse effects of this group are hyperkalemia, renal dysfunction, cough, and first-dose hypotension [29]. However, it is debatable whether it is safe to use ACE inhibitors and ARBs in hypertensive patients with COVID-19 [30]. On the one hand, ACE inhibitors and ARBs might upregulate ACE-2 expression, a cellular receptor for SARS-CoV-2. On the other hand, these drugs were shown to play a protective role in acute lung injury [31].

In this section, we reviewed studies that discussed the effects of ACE inhibitors and ARBs on the patients with hypertension in the context of the COVID-19 pandemic (Tables 1 and 2).

#### 1.4.1. Studies with Beneficial Effects

Data showing the beneficial effects of ACE inhibitors and ARBs in the treatment of hypertension during the COVID-19 pandemic are rising.

Several studies indicated that angiotensin II plays a crucial role in organ damage and production of various proinflammatory cytokines such as interleukin (IL)-1*β* and tumor necrosis factor (TNF)-*α* [28]. Furthermore, there is a positive association between elevated angiotensin II levels and lung injury in COVID-19 patients [32]. Therefore, the blockage of RAAS might be beneficial to prevent end-organ damage and inflammatory storm in COVID-19.

SARS-CoV-2 binds to endothelial and alveolar type 2 epithelial cells that express ACE-2 to a high degree, penetrates the cells via the activation of proteases such as transmembrane protease serine subtype 2 (TMPRSS2), infects these cells, and downregulates the expression of ACE-2 and thereby the ACE-2/Ang (1–7)/Mas-receptors (MasR) signaling pathway [33].

ACE-2 is an enzyme which breaks down angiotensin II into angiotensin 1–7 (Ang 1–7) [34]. Ang (1–7) exerts various effects, such as reducing the formation of myofibroblasts, collagen synthesis, and pulmonary fibrosis via MasR signaling pathways that have shown vasodilatory, anti-inflammatory, antiproliferative, and antifibrotic properties. This pathway also reduces the harmful effects of ACE-1-Ang II-AT1 receptor axis and at the same time enhances the ACE-2 activity. As a result, ACE-2 captures the S-proteins of SARS-CoV-2 in the plasma and thus prevents the virus from binding to lung cells [33, 35].

Although ACE-2 expression decreases after infection by SARS-CoV-2, numerous studies suggested that ACE inhibitors and ARBs increase the expression of ACE-2 and the plasma levels of Ang (1–7) [35]. It is noteworthy that no clinical data on the effects of ARBs and ACE inhibitors on ACE-2 expression in the lungs are available in either animal or human models [36].

As mentioned earlier, the dysregulation of RAAS due to the increased angiotensin II and decreased ACE-2 can lead to a severe inflammatory response. ACE inhibitors and ARBs may have a beneficial role in preventing these harmful effects by reducing angiotensin II and upregulating ACE-2 [28].

Several studies reported that COVID-19 patients with hypertension using ACE inhibitors/ARBs had a lower mortality rate, the risk of ICU admission, maximal viral load, the need for mechanical ventilation, and also decreased concentrations of high sensitivity C-reactive protein (hs-CRP), procalcitonin, and IL-6. Furthermore, they showed improved clinical outcomes in this population [31, 37–50].

Two other studies concluded that using ACE inhibitors/ARBs was associated with a significantly lower risk of hospitalization in hypertensive patients infected by SARS-CoV-2 [51, 52]. Furthermore, prehospitalization and in-hospital use of ACE inhibitors and ARBs also had a protective role in treating this population [53, 54]. In Tian et al.'s study, the discontinuation of ACE inhibitors and ARBs resulted in longer hospital stays [55]. In general, ACE inhibitors and ARBs should not be discontinued in hypertensive patients during the COVID-19 pandemic [47, 56].

#### 1.4.2. Studies with No Adverse Effects

Regarding the use of RAAS inhibitors in COVID-19 patients, some studies have not reported any adverse events in hypertensive patients with COVID-19 [57–59].

It was shown that there is no association between using ACE inhibitors/ARBs and the severity, mortality, clinical outcome, or poor prognosis of COVID-19 in the patients with hypertension [60–65].

Sardu et al. found no significant differences in the study endpoints including ICU admission, mechanical ventilation, cardiac injury, and deaths between patients in ACE inhibitors, ARBs, and CCBs groups [66]. Khera et al. reported that the use of ACE inhibitors and ARBs did not increase the risk of hospitalization or mortality in individuals with hypertension [67]. Furthermore, a large cohort study also found no association between using ACE inhibitors and ARBs and an increased likelihood of COVID-19 infection [68].

### 1.5. Spironolactone

Spironolactone is a pharmacological antagonist of aldosterone acting as a diuretic and an antihypertensive agent [69]. The most common side effects related to using spironolactone are lethargy, headache, ataxia, dyspepsia, nausea, vomiting, and anorexia [70].

Spironolactone is considered a safe option in COVID-19. Due to the increase in circulating ACE-2 and prevention of SARS-CoV-2 cell entry, spironolactone has a potential protective role in COVID-19 infection [71]. However, this drug has no effects on ACE-2 levels in the lungs [72]. Spironolactone can downregulate androgen-mediated TMPRSS2, which plays an important role in virus activation [71, 73, 74]. Besides, it can prevent pulmonary complications of COVID-19 through its anti-inflammatory and antiviral effects [71]. Spironolactone is effective in reducing SARS-CoV-2 infectivity, inhibit cytokine storm, and alleviate organ injury. Therefore, it can protect at all stages of COVID-19 infection [75].

### 1.6. Diuretics

Diuretics are among the most commonly prescribed agents used for the management of hypertension [4]. An association between using diuretics and several side effects including hyponatremia, hypokalemia, hypomagnesemia, acid-base changes, and hyperuricemia has been reported [76].

In contrast to other antihypertensive drugs, it was suggested that diuretics could have deleterious effects on cardiopulmonary interactions in COVID-19 patients receiving mechanical ventilation [77]. Furthermore, an association between using diuretics and a higher risk of cardiac injuries in COVID-19 has been observed [54].

### 1.7. Vasodilators and Central-Acting Agents

To date, no study was performed on the effects of vasodilators and central-acting agents on patients with hypertension and COVID-19.

### 1.8. Direct Renin Inhibitors

Direct renin inhibitors are a class of drugs used to block the effects of renin-angiotensin system [78]. Several adverse events, such as diarrhea, dizziness, fatigue, dry cough, and headache, were reported using aliskiren [79].

Oliveira et al. suggested that aliskiren could play an important role in preventing angiotensin I and II formation by direct blockade of renin and might have beneficial effects in the COVID-19 setting. However, they are associated with decreased ACE-2 expression with less infection gravity [80]. Furthermore, aliskiren was shown to be effective and safe for treating severe COVID-19 patients with hypertension [81].

## 2. Discussion

Patients with hypertension are at increased risk of severe COVID-19 infection and mortality [82]. The high prevalence of COVID-19 in the patients with preexisting hypertension raises major concerns about using antihypertensive drugs in such a population [83].

Yuan et al. reported that COVID-19 patients with hypertension taking RAAS inhibitors had a lower risk of death, ICU admissions, and septic shock, and patients who used beta-blockers, CCBs, and diuretics did not show any significant difference compared with the patients with uncontrolled blood pressure [54]. Besides, the patients in the ACE inhibitors and ARBs group had lower IL-6 levels and peak viral load than patients treated with beta-blockers, CCBs, and diuretics [43]. In a detailed meta-analysis, Ren et al. also confirmed that the mortality and severity of COVID-19 were significantly lower in the patients taking ACE inhibitors/ARBs than in controls. No association was found between using other antihypertensive drugs including CCBs, beta-blockers, and diuretics and the incidence and severity of COVID-19 [83]. However, Yan et al. suggested that antihypertensive drugs including ARBs, ACE inhibitors, CCBs, and beta-blockers, except for thiazide diuretics, might be beneficial for COVID-19 patients with hypertension [84].

In addition, previous studies have reported that the prevalence of COVID-19 infection differs between men and women [85]. A systematic review conducted by Abate et al. showed that the prevalence of COVID-19 in males and females was 55 and 45, respectively [86]. In several other studies, the rate of this infection was found to be higher in men than in women [87, 88]. Similarly, the male gender comprised the majority of hospitalized patients affected by COVID-19 [89, 90]. Moreover, among the hypertensive population with COVID-19, most patients have been reported to be males [89, 90].

Studies have suggested that the sex disparity of COVID-19-related morbidity and mortality is attributed to the differences in chromosomes and sex steroids, more engagement of men in activities such as smoking and alcohol consumption, and higher rates of comorbidities including hypertension and chronic obstructive pulmonary disease (COPD) in males [91]. According to these explanations, the gender might also affect the rate of response to hypertension treatment.

In contrast to the other studies, we investigated the effects of antihypertensive drugs on COVID-19 solely in the population of patients with hypertension. Recent data suggest that RAAS inhibitors have beneficial effects such as lower mortality and severity of COVID-19 infection in hypertensive patients [83]. Therefore, when compared to other antihypertensive drugs, ACE inhibitors and ARBs may be better choices to treat hypertension in this population. Conversely, diuretics can be considered the least effective drug in the setting of concomitant hypertension and COVID-19.

## 3. Conclusions

Based on the available literature, it is recommended to continue using antihypertensive agents in the patients with coexisting COVID-19 and hypertension. Furthermore, RAAS inhibitors may have superior beneficial effects to treat hypertension among this population. Further clinical studies on both genders are needed to validate the findings of this review.

## Figures and Tables

**Table 1:** Studies that showed neutral effects of ACEIs/ARBs on COVID-19 in hypertensive patients.

Study	Country	Drug group	Number of cases	Findings
Gao et al. [57]	China	RAAS inhibitors group	183	No harm
		Non-RAAS inhibitors group	527	

Ran et al. [58]	China	ARBs group	100	No increase in the risk of adverse events
		Other antihypertensive drugs	813	

Mustafic et al. [59]	France	Hypertensive patients with ACEIs or ARBs use	946	No difference in outcome
		Hypertensive patients without ACEI/ARB use	2035	

Haroon et al. [60]	UK	RAAS inhibitor group	29518	No association with all-cause mortality
		CCBs group	18895	

Li et al. [63]	China	Total patients with hypertension	362	No association with the severity or mortality
		ACEI/ARBs group	115	

Liu et al. [64]	China	ACEIs/ARBs group	74	No influence on increasing the severe form of COVID-19 infection
		CCBs group	83	

Hu et al. [61]	China	ACEIs/ARBs group	65	No influence on the severity and clinical outcome
		Non-ACEIs/ARBs group	84	

Wang et al. [65]	China	Hypertensive patients taking ACEIs/ARBs	315	No increased risk of developing severe COVID-19
		Nonhypertensive patients	308	

Kim et al. [62]	Korea	ACEIs/ARBs group	331	No association with COVID-19 severity
		Non-ACEIs/ARBs group	1580	

Sardu et al. [66]	Italy	ACEIs/ARBs group	45	No influence on the prognosis
		CCBs group	17	

Khera et al. [67]	USA	Hypertensive patients with the use of at least one antihypertensive drug	2263	No association with the risk of hospitalization or mortality
An et al. [68]	USA	ACEIs/ARBs group	8351	No increased likelihood of COVID-19 infection
		Other groups	8547	

**Table 2:** Studies that showed beneficial effects of ACEIs/ARBs on COVID-19 in hypertensive patients.

Study	Country	Drug groups	Number of cases	Findings
Guo et al. [31]	China	Hypertensive patients in ACEIs/ARBs and non-ACEIs/ARBs treatment groups	3936	Lower mortality rate
Megaly et al. [42]	USA	ACEIs/ARBs group	534	Lower mortality rate
		Non-ACEIs/ARBs group	2733	

Salah et al. [46]	USA, Italy	Total patients with hypertension	16101	Lower mortality rate
		Patients taking ACEIs or ARBs	7816	

Wang et al. [47]	China	ACEIs/ARBs group	8104	Lower risk of mortality and ventilatory support
		Non-ACEIs/ARBs group	8203	

Zhang et al. [50]	China	ACEIs/ARBs group	188	Lower mortality rate
		Non-ACEIs/ARBs group	940	

Negreira-Caamaño et al. [45]	Spain	Previous treatment with ACEIs/ARBs	392	Lower mortality rate
		No previous treatment with ACEIs/ARBs	153	

Desai et al. [39]	Italy	Total patients	575	Reduced mortality rate in chronic ACEIs users
		ACEIs or ARBs users	154	

Bae et al. [37]	Korea	RAAS inhibitors users	1076	Lower risk of ICU admission
		Never-users of RAAS inhibitors	298	

Yang et al. [49]	China	ACEIs/ARBs group	43	Lower concentrations of hs-CRP and procalcitonin
		Non-ACEIs/ARBs group	83	
		Controls	125	

Meng et al. [43]	China	ACEIs/ARBs group	17	Decreased IL-6 levels and peak viral load
		Non-ACEIs/ARBs group	25	

Barochiner et al. [38]	Argentina	Hypertensive patients taking ACEIs or ARBs	8328	Lower risk of death, admission to ICU, and mechanical ventilation
		Under other or no treatment	8983	

Lam et al. [41]	USA	Hypertensive patients	614	Lower ICU admission rate and mortality rate
Meng et al. [44]	China	Hypertensive patients in ACEIs/ARBs group	73	Improved clinical outcome such as lower death rate
		Non-ACEIs/ARBs group	186	

Kim et al. [40]	Korea	ACEIs/ARBs users	682	Lower clinical outcomes
		Non-users of ACEIs/ARBs	603	

Yuan et al. [54]	China	ACEIs/ARBs group	196	Protective effects
		Uncontrolled group	233	

Golpe et al. [51]	Spain	Hypertensive patients with hospital admission	69	Lower risk of hospitalization
		Outpatient management	88	

Semenzato et al. [52]	France	ACEIs/ARBs group	1524250	Lower risk of hospitalization and intubation
		CCBs group	358306	

Tian et al. [55]	China	Discontinued ACEIs/ARBs group	27	Discontinued ACEIs/ARBs group had longer hospital stays
		Other antihypertensive drugs group	26	

Chen et al. [53]	China	RAAS inhibitors group	355	Protective effects on mortality
		Non-RAAS inhibitors group	827	

ACEIs: angiotensin-converting enzyme inhibitors; ARBs: angiotensin II receptor blockers; hs-CRP: high-sensitivity C-reactive protein; IL-6: interleukin 6; ICU: intensive care unit; RAAS: renin-angiotensin-aldosterone system; CCBs: calcium channel blockers.

## Data Availability

Data sharing is not applicable to this article as no datasets were generated or analysed during the current study.

## References

[1] Staessen J. A., Wang J., Bianchi G., Birkenhäger W. H. (2003). Essential hypertension. *The Lancet*.

[2] Mills K. T., Bundy J. D., Kelly T. N. (2016). Global disparities of hypertension prevalence and control. *Circulation*.

[3] Fryar C. D., Ostchega Y., Hales C. M., Zhang G., Kruszon-Moran D. (2017). Hypertension prevalence and control among adults: United States, 2015–2016. *NCHS Data Brief*.

[4] Jackson R., Bellamy M. (2015). Antihypertensive drugs. *BJA Education*.

[5] Khalil H., Zeltser R. (2020). *Antihypertensive Medications*.

[6] Ruscitti P., Berardicurti O., Di Benedetto P., Cipriani P., Iagnocco A., Shoenfeld Y. (2020). Severe COVID-19, another piece in the puzzle of the hyperferritinemic syndrome. an immunomodulatory perspective to alleviate the storm. *Frontiers Immunology*.

[7] Carfora V., Spiniello G., Ricciolino R. (2020). Anticoagulant treatment in COVID-19: a narrative review. *Journal of Thrombosis and Thrombolysis*.

[8] Fang L., Karakiulakis G., Roth M. (2020). Are patients with hypertension and diabetes mellitus at increased risk for COVID-19 infection?. *The Lancet Respiratory Medicine*.

[9] Marin G. H. (2020). Facts and reflections on COVID-19 and anti-hypertensives drugs. *Drug Discoveries & Therapeutics*.

[10] Barrera F. J., Shekhar S., Wurth R. (2020). Prevalence of diabetes and hypertension and their associated risks for poor outcomes in Covid-19 Patients. *Journal of the Endocrine Society*.

[11] Whelton P. K., Carey R. M., Aronow W. S., Casey D. E., Collins K. J., Dennison Himmelfarb C. (2018). 2017 ACC/AHA/AAPA/ABC/ACPM/AGS/APhA/ASH/ASPC/NMA/PCNA guideline for the prevention, detection, evaluation, and management of high blood pressure in adults: a report of the American college of cardiology/American heart association task force on clinical practice guidelines. *Hypertension*.

[12] Hiremath S., Ruzicka M., Petrcich W. (2019). Alpha-blocker use and the risk of hypotension and hypotension-related clinical events in women of advanced age. *Hypertension*.

[13] Gibson P. G., Qin L., Puah S. H. (2020). COVID ‐19 acute respiratory distress syndrome (ARDS): clinical features and differences from typical pre‐ COVID ‐19 ARDS. *Medical Journal of Australia*.

[14] Koenecke A., Powell M., Xiong R. (2020). Alpha-1 adrenergic receptor antagonists to prevent hyperinflammation and death from lower respiratory tract infection. https://arxiv.org/abs/2004.10117.

[15] Farzam K., Jan A. (2020). Beta blockers.

[16] Chockalingam P., Crotti L., Girardengo G. (2012). Not all beta-blockers are equal in the management of long QT syndrome types 1 and 2. *Journal of the American College of Cardiology*.

[17] Vasanthakumar N. (2020). Beta-adrenergic blockers as a potential treatment for COVID-19 patients. *Bioessays*.

[18] Bauer A. Z., Gore R., Sama S. R. (2021). Hypertension, medications, and risk of severe COVID‐19: a Massachusetts community‐based observational study. *The Journal of Clinical Hypertension*.

[19] Kjeldsen S. E., Narkiewicz K., Burnier M., Oparil S. (2021). Potential protective effects of antihypertensive treatments during the Covid-19 pandemic: from inhibitors of the renin-angiotensin system to beta-adrenergic receptor blockers. *Blood Pressure*.

[20] Reynolds H. R., Adhikari S., Pulgarin C. (2020). Renin-angiotensin-aldosterone system inhibitors and risk of Covid-19. *New England Journal of Medicine*.

[21] Armstrong C. (2014). JNC8 guidelines for the management of hypertension in adults. *American Family Physician*.

[22] McKeever R. G., Hamilton R. J. (2020 2020). *Calcium Channel Blockers*.

[23] Webb R. C. (2003). Smooth muscle contraction and relaxation. *Advances in Physiology Education*.

[24] Russell R. P. (1988). Side effects of calcium channel blockers. *Hypertension (Dallas, Tex: 1979)*.

[25] Zhang L. K., Sun Y., Zeng H. (2020). Calcium channel blocker amlodipine besylate therapy is associated with reduced case fatality rate of COVID-19 patients with hypertension. *Cell Discovery*.

[26] Solaimanzadeh I. (2020). Nifedipine and amlodipine are associated with improved mortality and decreased risk for intubation and mechanical ventilation in elderly patients hospitalized for COVID-19. *Cureus*.

[27] Nouri‐Vaskeh M., Kalami N., Zand R. (2021). Comparison of losartan and amlodipine effects on the outcomes of patient with COVID‐19 and primary hypertension: a randomized clinical trial. *International Journal of Clinical Practice*.

[28] Wang J. J., Edin M. L., Zeldin D. C., Li C., Wang D. W., Chen C. (2020). Good or bad: application of RAAS inhibitors in COVID-19 patients with cardiovascular comorbidities. *Pharmacology & Therapeutics*.

[29] Alderman C. P. (1996). Adverse effects of the angiotensin-converting enzyme inhibitors. *Annals of Pharmacotherapy*.

[30] Zhou P., Yang X. L., Wang X. G. (2020). A pneumonia outbreak associated with a new coronavirus of probable bat origin. *Nature*.

[31] Guo X., Zhu Y., Hong Y. (2020). Decreased mortality of COVID-19 with renin-angiotensin-aldosterone system inhibitors therapy in patients with hypertension: a meta-analysis. *Hypertension*.

[32] Liu Y., Yang Y., Zhang C. (2020). Clinical and biochemical indexes from 2019-nCoV infected patients linked to viral loads and lung injury. *Science China Life Sciences*.

[33] Rossi G. P., Sanga V., Barton M. (2020). Potential harmful effects of discontinuing ACE-inhibitors and ARBs in COVID-19 patients. *Elife*.

[34] Dhaundiyal A., Kumari P., Jawalekar S. S., Chauhan G., Kalra S., Navik U. (2020). Is highly expressed ACE 2 in pregnant women “a curse” in times of COVID-19 pandemic?. *Life Sciences*.

[35] Elshafei A., Khidr E. G., El-Husseiny A. A., Gomaa M. H. (2021). RAAS, ACE2 and COVID-19; a mechanistic review. *Saudi Journal of Biological Sciences*.

[36] Kai H., Kai M. (2020). Interactions of coronaviruses with ACE2, angiotensin II, and RAS inhibitors-lessons from available evidence and insights into COVID-19. *Hypertension Research*.

[37] Bae J. H., Choi S. K., Kim N. H., Lee J., Kim S. G. (2021). Use of renin-angiotensin-aldosterone system inhibitors and severe COVID-19 outcomes in patients with hypertension: a nationwide cohort study. *Korean Diabetes Journal*.

[38] Barochiner J., Martínez R. (2020). Use of inhibitors of the renin‐angiotensin system in hypertensive patients and COVID‐19 severity: a systematic review and meta‐analysis. *Journal of Clinical Pharmacy and Therapeutics*.

[39] Desai A., Voza G., Paiardi S. (2021). The role of anti-hypertensive treatment, comorbidities and early introduction of LMWH in the setting of COVID-19: a retrospective, observational study in Northern Italy. *International Journal of Cardiology*.

[40] Kim J. H., Baek Y. H., Lee H., Choe Y. J., Shin H. J., Shin J. Y. (2021). Clinical outcomes of COVID-19 following the use of angiotensin-converting enzyme inhibitors or angiotensin-receptor blockers among patients with hypertension in Korea: a nationwide study. *Epidemiol Health*.

[41] Lam K. W., Chow K. W., Vo J. (2020). Continued in-hospital angiotensin-converting enzyme inhibitor and angiotensin II receptor blocker use in hypertensive COVID-19 patients is associated with positive clinical outcome. *The Journal of Infectious Diseases*.

[42] Megaly M., Glogoza M. (2020). Renin-angiotensin system antagonists are associated with lower mortality in hypertensive patients with COVID-19. *Scottish Medical Journal*.

[43] Meng J., Xiao G., Zhang J. (2020). Renin-angiotensin system inhibitors improve the clinical outcomes of COVID-19 patients with hypertension. *Emerging Microbes & Infections*.

[44] Meng X., Liu Y., Wei C. (2020). Angiotensin converting enzyme inhibitors and angiotensin receptor blockers improved the outcome of patients with severe COVID-19 and hypertension. *Science China Life Science*.

[45] Negreira-Caamaño M., Piqueras-Flores J., Martínez-DelRio J. (2020). Impact of treatment with renin–angiotensin system inhibitors on clinical outcomes in hypertensive patients hospitalized with COVID-19. *High Blood Press Cardiovascular Prevention*.

[46] Salah H. M., Calcaterra G., Mehta J. L. (2020). Renin-angiotensin system blockade and mortality in patients with hypertension and COVID-19 infection. *Journal of Cardiovascular Pharmacology and Therapeutics*.

[47] Wang Y., Chen B., Li Y. (2020). The use of renin–angiotensin–aldosterone system (RAAS) inhibitors is associated with a lower risk of mortality in hypertensive COVID‐19 patients: a systematic review and meta‐analysis. *Journal of Medical Virology*.

[48] Wang Y., Chen B., Li Y. (2021). The use of renin-angiotensin-aldosterone system (RAAS) inhibitors is associated with a lower risk of mortality in hypertensive COVID‐19 patients: a systematic review and meta‐analysis. *Journal of Medical Virology*.

[49] Yang G., Tan Z., Zhou L. (2020). Effects of angiotensin II receptor blockers and ACE (angiotensin-converting enzyme) inhibitors on virus infection, inflammatory status, and clinical outcomes in patients with COVID-19 and hypertension. *Hypertension*.

[50] Zhang P., Zhu L., Cai J. (2020). Association of inpatient use of angiotensin-converting enzyme inhibitors and angiotensin II receptor blockers with mortality among patients with hypertension hospitalized with COVID-19. *Circulation Research*.

[51] Golpe R., Pérez-de-Llano L. A., Dacal D., Guerrero-Sande H., Pombo-Vide B., Ventura-Valcárcel P. (2020). Risk of severe COVID-19 in hypertensive patients treated with renin-angiotensin-aldosterone system inhibitors. *Medicina Clínica*.

[52] Semenzato L., Botton J., Drouin J. (2021). Antihypertensive drugs and COVID-19 risk. *Hypertension*.

[53] Chen C., Wang F., Chen P. (2020). Mortality and pre‐hospitalization use of renin‐angiotensin system inhibitors in hypertensive COVID‐19 patients. *Journal of American Heart Association*.

[54] Yuan Y., Liu D., Zeng S. (2020). In-hospital use of ACEI/ARB is associated with lower risk of mortality and critic illness in COVID-19 patients with hypertension. *Journal of Infection*.

[55] Tian C., Li N., Bai Y. (2021). Angiotensin converting enzymes inhibitors or angiotensin receptor blockers should be continued in COVID-19 patients with hypertension. *World Journal of Clinical Cases*.

[56] Vila‐Corcoles A., Satue‐Gracia E., Ochoa‐Gondar O. (2020). Use of distinct anti‐hypertensive drugs and risk for COVID‐19 among hypertensive people: a population‐based cohort study in Southern Catalonia, Spain. *Journal of Clinical Hypertension*.

[57] Gao C., Cai Y., Zhang K. (2020). Association of hypertension and antihypertensive treatment with COVID-19 mortality: a retrospective observational study. *European Heart Journal*.

[58] Ran J., Song Y., Zhuang Z. (2020). Blood pressure control and adverse outcomes of COVID-19 infection in patients with concomitant hypertension in Wuhan, China. *Hypertension Research*.

[59] Mustafic H., Fayssoil A., Josseran L. (2021). Impact of angiotensin-converting enzyme inhibitors and angiotensin ii receptor blockers in hypertensive patients with COVID-19 (COVIDECA study). *The American Journal of Cardiology*.

[60] Haroon S., Subramanian A., Cooper J. (2021). Renin-angiotensin system inhibitors and susceptibility to COVID-19 in patients with hypertension: a propensity score-matched cohort study in primary care. *BMC Infectious Diseases*.

[61] Hu J., Zhang X., Zhang X. (2020). COVID-19 is more severe in patients with hypertension; ACEI/ARB treatment does not influence clinical severity and outcome. *The Journal of Infection*.

[62] Kim H. S., Kang M., Kang G. (2021). Renin-angiotensin system modulators and other risk factors in COVID-19 patients with hypertension: a Korean perspective. *BMC Infectious Diseases*.

[63] Li J., Wang X., Chen J., Zhang H., Deng A. (2020). Association of renin-angiotensin system inhibitors with severity or risk of death in patients with hypertension hospitalized for coronavirus disease 2019 (COVID-19) infection in Wuhan, China. *JAMA Cardiology*.

[64] Liu X., Liu Y., Chen K. (2020). Efficacy of ACEIs/ARBs vs CCBs on the progression of COVID‐19 patients with hypertension in Wuhan: a hospital‐based retrospective cohort study. *Journal of Medical Virology*.

[65] Wang S., Zhang Q., Wang P. (2021). Clinical features of hypertensive patients with COVID-19 compared with a normotensive group: single-center experience in China. *Open Medicine*.

[66] Sardu C., Maggi P., Messina V. (2020). Could anti‐hypertensive drug therapy affect the clinical prognosis of hypertensive patients with COVID‐19 infection? data from centers of southern Italy. *Journal American Heart Association*.

[67] Khera R., Clark C., Lu Y. (2020). Association of angiotensin-converting enzyme inhibitors and angiotensin receptor blockers with the risk of hospitalization and death in hypertensive patients with coronavirus disease-19. *medRxiv*.

[68] An J., Wei R., Zhou H. (2021). Angiotensin‐converting enzyme inhibitors or angiotensin receptor blockers use and COVID‐19 infection among 824 650 patients with hypertension from a US integrated healthcare system. *Journal American Heart Association*.

[69] Gupta A., Indurkhya A., Chaturvedi S., Varma A. (2019). Formulation and characterization of self emulsifying drug delivery system of spironolactone. *Asian Journal of Pharmaceutical Research and Development*.

[70] Carone L., Oxberry S. G., Twycross R., Charlesworth S., Mihalyo M., Wilcock A. (2017). Spironolactone. *Journal of Pain and Symptom Management*.

[71] Cadegiani F. A., Wambier C. G., Goren A. (2020). Spironolactone: an anti-androgenic and anti-hypertensive drug that may provide protection against the novel coronavirus (SARS-CoV-2) induced acute respiratory distress syndrome (ARDS) in COVID-19. *Frontiers in Medicine*.

[72] Cadegiani F. A., Wambier C. G., Goren A. (2020). Spironolactone: an anti-androgenic and anti-hypertensive drug with strong potential to prevent Covid-19 induced acute respiratory distress syndrome (ARDS). *Frontiers in Medicine*.

[73] Mollica V., Rizzo A., Massari F. (2020). The pivotal role of TMPRSS2 in coronavirus disease 2019 and prostate cancer. *Future Oncology*.

[74] Liaudet L., Szabo C. (2020). Blocking mineralocorticoid receptor with spironolactone may have a wide range of therapeutic actions in severe COVID-19 disease. *Critical Care (London, England)*.

[75] Cadegiani F. A., Goren A., Wambier C. G. (2020). Spironolactone may provide protection from SARS-CoV-2: targeting androgens, angiotensin converting enzyme 2 (ACE2), and renin-angiotensin-aldosterone system (RAAS). *Medical Hypotheses*.

[76] Sica D. A. (2004). Diuretic‐related side effects: development and treatment. *The Journal of Clinical Hypertension*.

[77] Tsolaki V., Zakynthinos G. E., Mantzarlis K., Makris D. (2020). Increased mortality among hypertensive COVID-19 patients: pay a closer look on diuretics in mechanically ventilated patients. *Heart & Lung*.

[78] Hammoud R. A., Vaccari C. S., Nagamia S. H., Khan B. V. (2007). Regulation of the renin–angiotensin system in coronary atherosclerosis: a review of the literature. *Vascular Health Risk Management*.

[79] Pich J. (2018). The efficacy of renin inhibitors in primary hypertension. *AJN, American Journal of Nursing*.

[80] de Oliveira G. M., Doria Rossi M. I. (2020). The possible management of hypertensive patients infected with COVID-19 through the use of aliskiren. *Nephrology journal and Renal Diseases*.

[81] Guo Y., Zeng J., Li Q. (2020). Preliminary clinical study of direct renin inhibitor aliskiren in the treatment of severe COVID-19 patients with hypertension. *Zhonghua nei ke za zhi*.

[82] Dalan R., Ang M. L. W., Tan W. Y. (2020). The association of hypertension and diabetes pharmacotherapy with COVID-19 severity and immune signatures: an observational study. *European Heart Journal Cardiovascular Pharmacotherapy*.

[83] Ren L., Yu S., Xu W., Overton J. L., Chiamvimonvat N., Thai P. N. (2020). Lack of association of antihypertensive drugs with the risk and severity of COVID-19: a meta-analysis. *Journal of Cardiology*.

[84] Yan F., Huang F., Xu J. (2020). Antihypertensive drugs are associated with reduced fatal outcomes and improved clinical characteristics in elderly COVID-19 patients. *Cell Discovery*.

[85] Ya’qoub L., Elgendy I. Y., Pepine C. J. (2021). Sex and gender differences in COVID-19: more to be learned. *American Heart Journal Plus: Cardiology Research and Practice*.

[86] Abate B. B., Kassie A. M., Kassaw M. W., Aragie T. G., Masresha S. A. (2020). Sex difference in coronavirus disease (COVID-19): a systematic review and meta-analysis. *BMJ Open*.

[87] Adams R. B. (2020). Gender equality in work and Covid-19 deaths. *Covid Economics*.

[88] Sun P., Lu X., Xu C., Sun W., Pan B. (2020). Understanding of COVID‐19 based on current evidence. *Journal of Medical Virology*.

[89] Abayomi A., Osibogun A., Kanma-Okafor O. (2021). Morbidity and mortality outcomes of COVID-19 patients with and without hypertension in Lagos, Nigeria: a retrospective cohort study. *Global Health Research Policy*.

[90] Zhong L., Wu Y., Gao J. (2021). Effects of hypertension on the outcomes of COVID-19: a multicentre retrospective cohort study. *Annals of Medicine*.

[91] Haitao T., Vermunt J. V., Abeykoon J. (2020). COVID-19 and sex differences: mechanisms and biomarkers. *Mayo Clinic Proceedings*.

